# Notes on Preparations for the Sick

**Published:** 1900-06

**Authors:** 


### Notes on Preparations for the Sick.

---

Many new food preparations have been devised of late. These foods are not only excellent aids to medicinal treatment, but are often the only treatment necessary or even possible. It has been pointed out recently by Mitchell Bruce that the practitioner must never lose control of the diet. It is not enough that he should permit different foods, he must employ them definitely as carefully-ordered means of treatment. And when he is deliberately planning treatment and reviewing his remedies in each case he should always think of food before he thinks of medicine, and give it a corresponding position of importance in his directions to the nurse or patient.

Dettweiler's dictum, "Ma cuisine c'est ma pharmacie," is the modern view as regards medication in phthisis, where systematic over-feeding has become the established rule, and in other diseases variations in the quantity of food, even to complete fasting, may be the best form of medication, but on the other hand "a great many persons are short of elimination, not of alimentation, when they are looking poorly. It is a fortunate circumstance that many so-called 'strengthening things' are really of low nutritive value and that others of them are very imperfectly assimilated." (Mitchell Bruce, *Principles of Treatment*, 1899, p. 201.)

It is unfortunate that many, and some of the most useful, food preparations are proprietary articles to which there usually attaches the stigma that they are prepared and sold chiefly for the benefit of the proprietor. This is no doubt a reason why so many of such food preparations, although taken largely by the patient under the influence of advertisement, are not commonly used by the physician as important means of treatment.

The numerous **Milk Preparations** now on the market are evidence of the now well-established belief that "milk from a cow with tuberculous disease of the udder is of all food products the most deadly. At whatever rate the danger from meat may be estimated, about that from tuberculous milk there can be only one opinion. The evidence is overwhelming." (Hillier, *Tuberculosis*, 1900, p. 103.)

"The risk which attaches to milk extends to all the products of milk in the preparation of which no process of heating is employed. Butter, skimmed milk, butter-milk, cream, and cheese may all contain tubercle bacilli if obtained from a contaminated milk." (*Ibid.*, p. 107.)

**The process of Pasteurisation** is now becoming recognised as the most satisfactory guarantee wherever the fresh milk is preferred to other manufactured products. "Pasteurisation, which is practically heating milk for some time at a temperature below actual boiling, and other modifications of this, such as heat under pressure, have the effect of sterilizing milk without depriving it of its butter-, cream-, and cheese-producing qualities." (*Ibid.*, p. 108.)

As long as tuberculosis exists amongst cattle the boiling, or the Pasteurisation, of cows milk is a precaution necessary in all kitchens and nurseries—in this way the danger from milk is capable of absolute control. Some dairymen give a guarantee that the tuberculin test shall be used periodically amongst all their cattle, but it is scarcely reasonable to expect that all dairymen shall act up to any such standard; it is quite open to question whether the Pasteurisation of milk and cream does not give a better security than can be obtained by any kind of selection of the animals.

The **process of peptonisation** of the milk usually comprises the boiling, and hence the sterilisation, of the product, but this is, of course, only one element in favour of artificial digestion when required.

One of the most satisfactory milk products is now sold as **Plasmon**—Nature's nutrient: soluble, digestible, natural albumen. (The Plasmon Syndicate, 56 Duke Street, W.) One teaspoonful of this powder contains the amount of nutriment equivalent to a quarter-pound of beef, or of about two pints of milk. It consists of the milk proteids in the form of a soluble dry powder—tasteless, odourless, and easily digested,—it is therefore a highly concentrated natural albumen, with no admixture of fat or farinaceous products. Merck, *Annual Report for 1899*, p. 126, describes it as "a mixture of albuminoids obtained from skimmed milk with a barely sufficient quantity of sodium bicarbonate to obtain complete solution. It is a slightly yellowish, odourless and tasteless gritty powder and soluble in warm water . . . Adults may be given up to 120.0 grm. (4 ounces) of plasmon per day either baked into bread or added to bouillon or broth without risk of discomfort." A concentrated powder of this kind, with no objectionable properties, may be taken in increasing doses, and should be of the greatest value in carrying out the process of over-feeding whenever this may be desirable. Plasmon is also sold in biscuits and chocolate tablets.

Another very nourishing food not new, but which (we are informed) has been recently modified and much improved, is sold by the Bovril Company as **Virol**. It contains the red bone marrow from ox-ribs, extracted by the glycerine process, and fresh eggs, of which the shells have been broken up and dissolved in lemon-juice, together with some malt extract. A modification of this, containing the yellow marrow of the long bones, is known as **Marrol**. Both these preparations have proved themselves to be valuable adjuncts to the diet table in many cases of defective nutrition, and more especially in cases of pronounced anæmia.

The **Somatose** group (Bayer's Food Products) have been noticed by us on several occasions—September, 1895, March and September, 1899. A highly concentrated meat albumen in a soluble form is a very different thing from ordinary meat extracts; the latter have no actual value in the work of tissue building, whereas somatose powder contains about 80 per cent. of albumose, and is in the highest degree capable of tissue renovation, with very little trouble to the organism by way of digestion and assimilation.—(The British Somatose Company, London, E.C.)

Another food—an insoluble one, not a milk product—is sold under the designation **Tropon** (Troponwerke: Mulheim am Rhein. Arthur Reiner & Co., Old Broad Street, E.C.). This substance is a dry powder, with little taste or odour. It is absolutely insoluble in water, and therefore contains no soluble albumen. After standing in water for many hours the powder remains unchanged at the bottom of the test tube, and the supernatent fluid gives no precipitate with heat or nitric acid. The powder is described as "albumen in its purest and most digestible form." This apparently means that it can be easily digested with pepsin and hydrochloric acid. It is not hygroscopic: it therefore will keep well, and it may be utilised in many ways, either by itself as a food or to increase the nutritive value of made dishes and fluid drinks. A pamphlet by Professor Finkler professes to give an exhaustive account of tropon as a food for invalids, from an experience of one hundred cases, and from it we learn that "Tropon will agree with everybody. It does not present any drawback either chemical or mechanical, for digestion or absorption. . . . We have laid great stress on administering tropon to invalids without their knowledge; this can be done by mixing it with soup, porridge, and most advantageously of all, with chocolate and cocoa. . . . The invalid must be convinced that albumen is not a medicine which contains a great amount of strength-producing properties in a very small bulk. He must be told that albumen is a food, which, however concentrated it may be, must be taken in considerable quantities and for some time, to produce the desired effects." It has been given in many diseases in quantities amounting to an average of 20 grams a day, and has given very good results. We are not informed from whence this food is derived, nor how it is prepared; microscopic examination throws no light on its origin, as we could not identify either muscle fibres or starch granules. One pound is said to be equivalent in nourishing value to five pounds of beef (without fat or bone) or from 85 to 100 eggs.

